# Venous Thromboembolism Management throughout the COVID-19 Era: Addressing Acute and Long-Term Challenges

**DOI:** 10.3390/jcm13061825

**Published:** 2024-03-21

**Authors:** Maddalena Alessandra Wu, Alba Taino, Pietro Facchinetti, Valentina Rossi, Diego Ruggiero, Silvia Berra, Giulia Blanda, Nicola Flor, Chiara Cogliati, Riccardo Colombo

**Affiliations:** 1 Division of Internal Medicine, ASST Fatebenefratelli Sacco, Luigi Sacco Hospital, University of Milan, 20156 Milan, Italy; 2 Department of Biomedical and Clinical Sciences, University of Milan, 20156 Milan, Italy; 3 Division of Emergency Medicine, ASST Fatebenefratelli Sacco, Luigi Sacco Hospital, 20157 Milan, Italy; 4 Division of Cardiology, ASST Fatebenefratelli Sacco, Luigi Sacco Hospital, 20157 Milan, Italy; 5 Department of Radiology, ASST Fatebenefratelli Sacco, Luigi Sacco Hospital, 20157 Milan, Italy; 6 Division of Anesthesiology and Intensive Care, ASST Fatebenefratelli Sacco, Luigi Sacco Hospital, University of Milan, 20156 Milan, Italy; riccardo.colombo@asst-fbf-sacco.it

**Keywords:** venous thromboembolism, pulmonary embolism, deep vein thrombosis, COVID-19, outcomes, management

## Abstract

**Background:** COVID-19 increases the risk of venous thromboembolism (VTE) through a complex interplay of mechanisms collectively referred to as immunothrombosis. Limited data exist on VTE challenges in the acute setting throughout a dynamic long-term follow-up of COVID-19 patients compared to non-COVID-19 patients. The aim of the study was to investigate acute and long-term management and complications in VTE patients with and without COVID-19. **Methods:** A prospective, observational, single-center cohort study on VTE patients followed from the acute care stage until 24 months post-diagnosis. **Results:** 157 patients, 30 with COVID-19-associated VTE and 127 unrelated to COVID-19, were enrolled. The mean follow-up was 10.8 (±8.9) months. COVID-19 patients had fewer comorbidities (1.3 ± 1.29 vs. 2.26 ± 1.68, *p* < 0.001), a higher proportion of pulmonary embolism at baseline (96.7% vs. 76.4%, *p* = 0.01), and had a lower probability of remaining on anticoagulant therapy after three months (*p* < 0.003). The most used initial therapy was low-molecular-weight heparin in 130/157 cases, followed by long-term treatment with direct oral anticoagulants in 123/157. Two (6.7%) COVID-19 vs. three (2.4%) non-COVID-19 patients (*p* = 0.243) had major hemorrhagic events, all of them within the first three months. Four (3.1%) non-COVID-19 patients had VTE recurrence after six months. Three (2.4%) non-COVID-19 patients developed chronic thromboembolic pulmonary hypertension. There were no fatalities among patients with COVID-19, compared to a mortality of 12/127 (9.4%) in the non-COVID-19 subgroup (*p* = 0.027). **Discussion:** Our study offers a comprehensive overview of the evolving nature of VTE management, emphasizing the importance of personalized risk-based approaches, including a limited course of anticoagulation for most COVID-19-associated VTE cases and reduced-dose extended therapy for high-risk subsets.

## 1. Introduction

Venous thromboembolism (VTE), encompassing deep vein thrombosis (DVT) and pulmonary embolism (PE), presents a considerable health challenge due to its multifactorial pathogenesis and potential short- and long-term complications. Despite its potential fatality, timely and appropriate anticoagulant therapy, along with vigilant patient monitoring, has led to a gradual reduction in mortality rates, highlighting the significance of comprehensive VTE management [1].

Beyond the risks of mortality and recurrence, paying attention to thromboembolic complications is of paramount importance. Recurrences are not the sole long-term complications. Post-thrombotic syndrome develops in 20–50% of patients with a history of DVT [2,3], while a wide range of incidences has been reported for chronic thromboembolic pulmonary hypertension (CTEPH), complicating from 0.1 up to more than 10% of PE events [4,5,6,7]. Establishing adequate follow-up is crucial for monitoring clinical status and identifying complications early, when they are still treatable. Some complications, like CTEPH, require not only further diagnostic work-up (e.g., cardiopulmonary tests, right heart catheterization) but may also necessitate invasive procedures, such as surgical intervention (endarterectomy) or interventional procedures (e.g., balloon pulmonary angioplasty), for which patients must be referred to specialized centers [7,8,9].

The COVID-19 pandemic introduced additional challenges, marked by complex mechanisms, including inflammatory response, endothelial dysfunction, and hemostatic abnormalities leading to COVID-19-associated coagulopathy or immunothrombosis [10,11,12,13]. This exacerbates the VTE challenge, with PE incidence ranging from 1% to 20% in hospitalized patients [14,15,16]. Autopsy studies confirm a high thrombotic burden in patients who succumbed to COVID-19, especially in severe cases or in those admitted to intensive care units, where additional factors, such as prolonged immobilization and respiratory failure, heighten VTE risks [17,18].

Numerous trials have explored treatments for micro and macrothrombotic complications due to COVID-19. However, limited data are available on the outcomes of VTE associated with COVID-19, especially in the long term. Guidelines recommend individualized risk stratification for thrombotic and hemorrhagic risks [19,20]. Still, the optimal anticoagulation strategy—whether short-term, extended, or indefinite—lacks robust evidence.

Proper risk stratification is imperative for selecting the most suitable anticoagulation regimen, considering the bleeding risks associated with anticoagulation [21,22,23]. The comprehensive management of VTE throughout the COVID-19 spectrum demands a nuanced approach, considering the multifactorial nature of the disease, the impact of SARS-CoV-2 infection, and the need for a dynamic evaluation of the balance between thrombotic and bleeding risk factors as patients recover from COVID-19.

The complexity of managing patients with VTE is evident and demands a well-organized and structured approach. In clinical practice, involving various specialist physicians who can collaborate from the outset toward a functional diagnostic and therapeutic pathway is inevitable, as suggested by the ESC guidelines [8,19]. This multi-professional team (including internists, emergency physicians, anesthesiologists, cardiologists, vascular surgeons, radiologists, hematologists, pulmonologists, and cardiothoracic surgeons) aims to enhance access to therapies, overcome management difficulties, and streamline the care of individual patients. The group approach also allows for the study of individual cases and the proposal of personalized therapeutic and management solutions.

The multidisciplinary approach, which has proven useful for treating acute thromboembolic events, also plays a significant role in managing long-term complications, such as CTEPH.

This study aimed to investigate acute management and long-term follow-up, focusing on the recurrence of venous thromboembolism, bleeding complications, and chronic thromboembolic pulmonary hypertension in a cohort of patients with VTE throughout the pandemic era of SARS-CoV-2.

## 2. Materials and Methods

This prospective observational cohort study enrolled adult patients diagnosed with VTE at the Luigi Sacco Hospital in Milan, Italy. The study started on 14 June 2019, and the analysis was conducted on data collected until 23 August 2023. The local Ethical Committee approved the study (Comitato Etico di Area 1, Milan, Italy, Protocol number 0025140/2023). Patients under 18 years of age were excluded.

Our study included patients diagnosed with venous thromboembolism, confirmed through both clinical assessment and instrumental investigations. Patients’ data were collected at baseline and at the following pre-specified time points: after 1, 3, 6, 12, 18, and 24 months.

Symptomatic PE was confirmed with a positive computed tomography (CT) angiography of the pulmonary arteries. DVT was confirmed using a compression ultrasound (CUS).

Predisposing factors for VTE in the studied population were categorized as major, moderate, and minor risk factors, according to the criteria reported by the ESC Guidelines on pulmonary embolism [19].

The revised Geneva and the simplified Pulmonary Embolism Severity Index (sPESI) were calculated at baseline. The VTE BLEED score was calculated at each time point.

The primary efficacy outcome was the rate of VTE recurrences within the first 12 months, and the primary safety outcome was major bleeding.

The secondary endpoints were the following: the number of subjects with presentation requiring thrombolysis and admission to the Intensive Care Unit; mortality; rate of thromboembolic-related or non-thromboembolic-related readmission; incidence of chronic thromboembolic pulmonary hypertension (CTEPH); incidence of clinically significant non-major bleeding events; and average duration of anticoagulant therapy.

The cohort was stratified according to VTE associated with COVID-19 or VTE unrelated to COVID-19. VTE was considered to be associated with COVID-19 if it occurred within 30 days of diagnosis of COVID-19. COVID-19 was diagnosed via a positive nasal swab for SARS-CoV-2 (with antigenic test or real-time PCR) or a positive real-time PCR on bronchial or alveolar fluids.

In patients with suspected VTE recurrences, the diagnosis was confirmed using a CT pulmonary angiography or compression ultrasound, as appropriate.

Major bleeding was defined according to the criteria of ISTH as clinically overt bleeding that was fatal or associated with any of the following: (a) a fall in hemoglobin level of 2 g/dL or more or documented transfusion of at least two units of packed red blood cells, (b) involvement of a critical anatomical site (intracranial, spinal, ocular, pericardial, articular, intramuscular with compartment syndrome, or retroperitoneal) [24].

Anticoagulant therapy received by the patient was recorded.

### Statistical Analysis

Descriptive statistics for categorical variables included counts and percentages, while mean and standard deviation (SD) were used for quantitative data. The Kolmogorov–Smirnov test was used to assess the normality of the data. Student’s *t* test or the Mann–Whitney U test were used to compare means between the two pre-specified subgroups, as appropriate. The Fisher’s exact test or the chi-square test were used to compare categorical variables. The Log-rank test was used to compare subgroups’ probability of remaining on anticoagulant therapy. *p* < 0.05 for a two-tailed test was considered statistically significant. Data analysis was conducted using IBM SPSS Statistics for Windows, Version 29.0 (IBM Corp., Armonk, NY, USA).

## 3. Results

In the study period, 157 patients were enrolled, 30 with VTE associated with COVID-19 and 127 with VTE unrelated to COVID-19. The mean follow-up time was 10.8 (±8.9) months. Patients’ characteristics are shown in Table 1. The mean age was 69.9 (±14.2) years, and 78 (49.7%) patients were male. COVID-19 patients, compared to non-COVID-19 patients, had fewer comorbidities (1.3 ± 1.29 vs. 2.26 ± 1.68, *p* < 0.001).

VTE was diagnosed in the emergency department in 102 (65%) patients, in the medical ward in 38 (24.2%), and during outpatient visits in 15 (9.6%) patients. Ten (6.4%) patients (four [13.3%] in the COVID-19 vs. six [4.7%] in the non-COVID-19 subgroup, *p* = 0.099) were admitted to the ICU.

Predisposing factors for VTE in the studied population did not differ between subgroups (Supplementary Table S1). Subgroups’ clinical characteristics at presentation are shown in Table 2.

COVID-19 patients had a higher prevalence of pulmonary embolism (*p* = 0.01), a lower prevalence of deep vein thrombosis (*p* < 0.001), and a lower prevalence of pulmonary embolism associated with deep vein thrombosis (*p* = 0.002). There were no differences between subgroups in the radiological characteristics of PE. Characterization of DVT by ultrasound at follow-up is shown in Supplementary Table S2.

The revised GENEVA risk was intermediate in 91 (58.71%) and high in 19 (12.26%) patients, with no difference between VTE associated with COVID-19 and VTE unrelated to COVID-19. Moreover, the simplified PESI was high in 84 (54.9%) patients, with no difference between the two subgroups.

Laboratory parameters at baseline did not differ between subgroups (Table 3).

Most patients in both subcategories were initially treated with low-molecular-weight heparin, followed by long-term therapy involving direct oral anticoagulants. Enoxaparin was the initial treatment used in 130 (79.6%) patients in the whole cohort (29 [96.7%] in the VTE associated with COVID-19 and 101 [79.5%] in the VTE unrelated to COVID-19 subgroups), followed by fondaparinux, which was used in 15 patients (1 [3.3%] COVID-19 patient and 14 [11%] non-COVID-19 patients, respectively), with no differences between subgroups in the choice of the first anticoagulant (*p* = 0.158) (Table 4).

Emergency thrombolysis was performed in three non-COVID-19 patients (2.4%) in the acute phase.

Long-term therapy was conducted with direct oral anticoagulants in 123 (78.3%) patients (25 [83.33%] in the VTE associated with COVID-19 vs. 98 [77.2%] in the VTE unrelated to COVID-19 subgroup).

**Figure 1:**
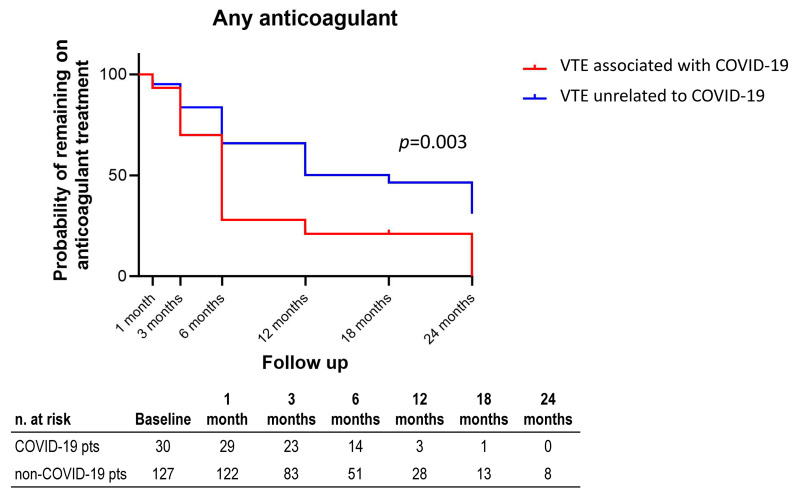
Probability of remaining on anticoagulant therapy during the follow-up. Patients with VTE associated with COVID-19 had, on average, a shorter duration of treatment compared to non-COVID-19 patients.

The probability of remaining on anticoagulant therapy differed between groups (*p* = 0.003), with a significantly lower proportion of VTE associated with COVID-19 patients continuing treatment after three months (Figure 1).

The risk of bleeding, assessed by VTE BLEED score, across the time points is shown in Supplementary Table S3. The proportion of patients at low risk differed between subgroups at six months (84.2% of COVID-19 vs. 55.6% of non-COVID-19, *p* = 0.0298).

Trans-thoracic echocardiographic data were available for 94 (59.9%) patients at the baseline (15 COVID-19 and 79 non-COVID-19). One (6.7%) patient with VTE associated with COVID-19 compared to sixteen (20.2%) patients with VTE unrelated to COVID-19 had echocardiographic signs of pulmonary hypertension (*p* = 0.291) (Supplementary Table S4).

Three non-COVID-19 patients developed chronic thromboembolic pulmonary hypertension (CTEPH), two of them diagnosed at three months and one at six-month follow-up. All three patients underwent cardiopulmonary exercise testing, right heart catheterization, and pulmonary endarterectomy (PEA), with a subsequent net improvement in their clinical picture and quality of life.

**Figure 2:**
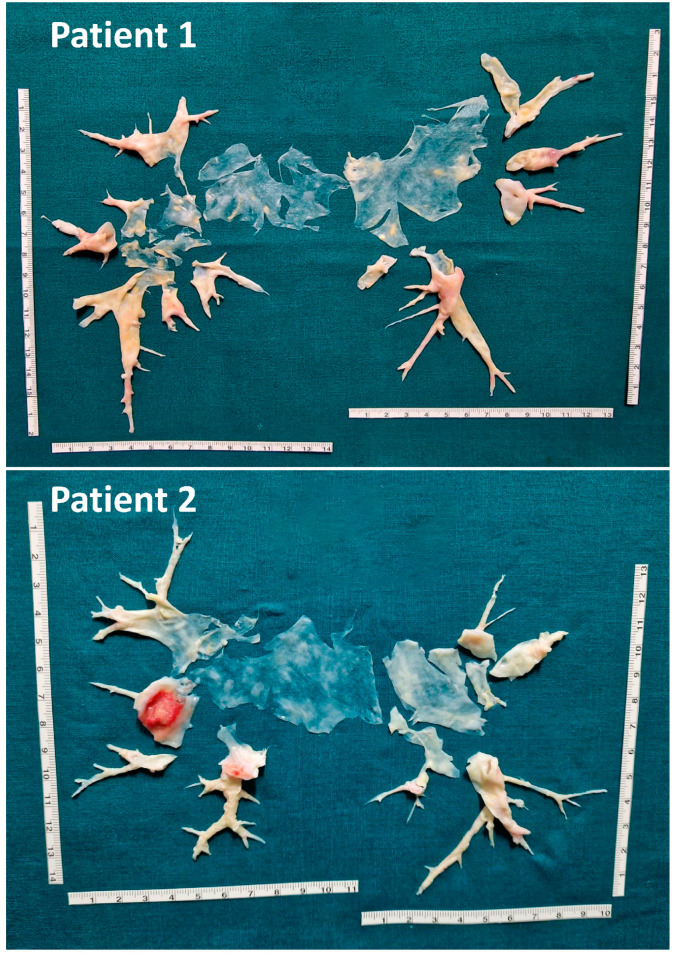

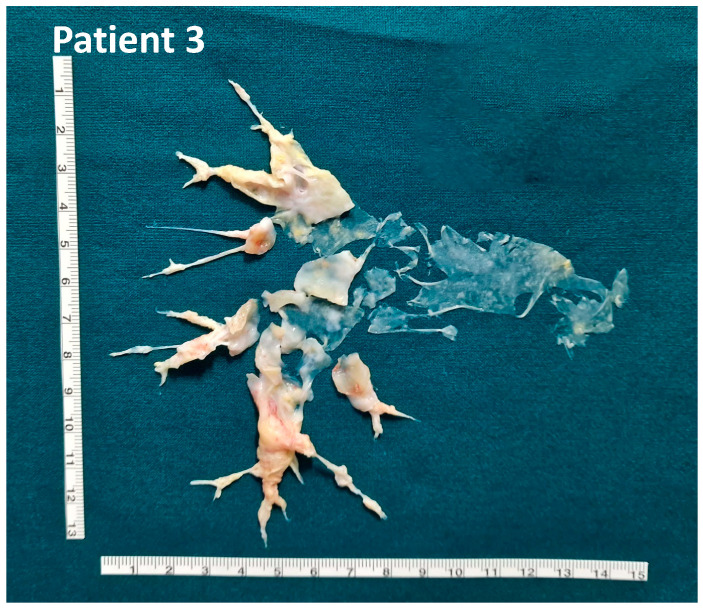
Intraoperative findings of pulmonary endarterectomy in the three subjects who developed chronic thromboembolic pulmonary hypertension (CTEPH).

Figure 2 shows PEA specimens.

Between 6 and 12 months, four non-COVID-19 patients experienced VTE recurrence in the form of DVT, with one case also involving PE. Five (3.2%) patients (two patients [6.7%] in the VTE associated with COVID-19 subgroup vs. three [2.4%] in the VTE unrelated to COVID-19 subgroup, *p* = 0.243) experienced six major hemorrhagic events, and seven (4.5%) patients (1 [3.3%] in the VTE associated with COVID-19 subgroup vs. 6 [4.7%] in the VTE unrelated to COVID-19 subgroup, *p* = 0.28) had nine clinically relevant non-major bleeding episodes during the study period. All hemorrhagic events occurred within the first three months.

Thirteen (8.3%) patients reported new hospitalization in the follow-up period, two of them because of VTE-related causes while the remaining episodes were due to non-VTE-related reasons.

There were no fatalities among patients with COVID-19, compared to a mortality of 12/127 (9.4%) in the non-COVID-19 subgroup (*p* = 0.027).

## 4. Discussion

Our study delves into the intricate realm of VTE, confirming the nuanced landscape presented by individuals with and without COVID-19. Through our investigation, we have unveiled similarities and distinctions between these two populations. Notably, the observation that pulmonary embolism predominantly occurs in COVID-19 patients without associated deep vein thrombosis aligns with the hypothesis of in situ thrombosis. This correlation is linked to the prothrombotic environment resulting from the cytokine storm induced by SARS-CoV-2, particularly at the pulmonary level.

Accumulating evidence suggests that VTE risk estimates in the COVID-19 population should be interpreted with caution. These estimates may suffer from various sources of heterogeneity, making it difficult to compare these patients even to those affected by other viral conditions, such as H1N1 influenza and SARS-CoV-1 [25]. At the core of our findings lies a delicate balance between recognizing the transient risk posed by COVID-19 and tailoring anticoagulation strategies to individual patient profiles.

Our investigation aligns with contemporary guidelines, affirming the safety of a finite course of anticoagulation (typically 3–6 months) for most patients with COVID-19-associated VTE [26]. The reassuringly rare occurrence of adverse events in our long-term follow-up, extending up to 24 months, underscores the credibility of perceiving SARS-CoV-2 infection as a transient risk factor. This perspective finds resonance in recent Italian research, revealing comparable medium- to long-term recurrence rates between patients treated for three months and those treated for extended periods [27]. Similarly, in the VTE unrelated to the COVID-19 population, we found some “provoked” cases in which it was possible to identify a transient risk factor (such as infections, surgeries, bed rest lasting for more than three days, or lower-limb fractures). Consequently, even in this group of patients, it has sometimes been possible to consider discontinuing therapy at the end of the 3–6 months of anticoagulant treatment.

However, our study emphasizes the imperative of early and nuanced risk stratification. While COVID-19 serves as a transient risk factor, it coexists with other potential risk factors, prompting a personalized assessment of therapy duration. For a subset of COVID-19 patients, particularly those with additional risk factors, we opted for a reduced-dose extended therapy approach beyond the initial six months.

Despite these “caveats”, the Kaplan–Meier analysis highlights a statistically significant difference in the duration of therapy in the two populations under study. In fact, in the COVID-19 category, the discontinuation of anticoagulant treatment was more frequent, starting from the third month after the index event. Importantly, this decision did not result in a significant increase in the recurrence of VTE or VTE-related rehospitalization outcomes in the medium to long term, reinforcing the appropriateness of the choice.

The intriguing aspect of our findings lies in the delicate interplay between thrombotic and bleeding risks.

While the reliability of bleeding scores is often questioned, our cohort exhibited no significant baseline differences in bleeding risk according to the VTE-BLEED score. The incidence of major and clinically relevant non-major bleeding events was remarkably low in both the COVID-19 and non-COVID-19 cohorts, in contrast to reports suggesting a higher bleeding rate in VTE associated with COVID-19 [28]. Notably, the rare bleeding events occurred in the first few months after starting anticoagulation therapy, a period known for elevated thrombotic and bleeding risk, making it particularly challenging to balance these factors.

Our diagnostic settings primarily comprised the Emergency Department and inpatient scenarios, including a spectrum of units such as Internal Medicine, Pneumology, Infectious Diseases, and Cardiology. The diagnosis was only occasionally confirmed in an outpatient setting, and this was limited to cases of VTE unrelated to COVID-19. Interestingly, possibly due to the specific working and structural setup of our Emergency Department, there were no acute cases managed in an Intensive Short-Term Observation setting. While this could also be attributed to the specific “labeling” of the area in which patients underwent prolonged multi-parameter monitoring (such as waiting areas, emergency cubicles, etc.), this element underscores the need for a more effective diagnostic–therapeutic and monitoring pathway, as commonly observed in numerous hospital and organizational settings. This pathway should enable the timely identification of patients who, despite not meeting baseline criteria for immediate discharge or clear indications for hospitalization, could benefit from close monitoring by the Emergency Department. Following this monitoring, any uncertainty regarding the possibility of discharge with territorial management can be resolved, leading either to redirection to the primary care physician or a decision for hospitalization.

Enoxaparin emerged as the predominant therapy, especially in emergency settings, in both COVID-19 and non-COVID-19 VTE patients. This preference may reflect the perceived pleiotropic effects of enoxaparin [29], coupled with a persistent hesitancy to adopt Direct Oral Anticoagulants (DOACs) in acute settings. However, our data indicate an early shift towards DOACs for long-term management in both categories, predominantly occurring from the first outpatient assessment onward, in alignment with contemporary recommendations.

Despite no statistically significant differences being highlighted at baseline between VTE patients with or without COVID-19, probably due to the limited sample size, we observed a trend towards a higher burden of comorbidities in non-COVID-19 patients. This provides insight into both the acute presentation and the long-term course of these patients. In this regard, in our cohort, patients with hemodynamic instability requiring thrombolysis were rare and exclusively represented in the VTE unrelated to COVID-19 subgroup. Recurrences of venous thromboembolism occurred in our cohort exclusively in the VTE unrelated to COVID-19 subgroup, likely characterized by an overall higher risk profile than COVID-19 patients (featuring the presence of multiple risk factors, permanent risk factors, or even so-called “unprovoked” thromboembolic events). On the other hand, it is equally significant that cases of chronic thromboembolic pulmonary hypertension (identified through multidimensional assessment and, subsequently, candidates for further diagnostic investigations as well as endarterectomy surgery) occurred exclusively in the population with VTE unrelated to COVID-19. In this context, it is interesting to note that in our cohort, two out of the three patients who developed chronic thromboembolic pulmonary hypertension, requiring endarterectomy, had a clinical course influenced by social–historical–cultural issues. In fact, in one of these two cases, the diagnosis of VTE was made in an African country where access to healthcare was limited, and it is possible that access to information about the condition and awareness of the importance of adequate and prolonged therapy were limited, resulting in poor therapeutic adherence. In the second case, the peak period of the COVID-19 emergency proved to be an obstacle to proper therapeutic management.

In our cohort, establishing a structured and integrated follow-up process contributed to high therapy adherence and improved quality of life.

In highlighting the strengths inherent in our study, the quite long follow-up period that extended beyond the conclusion of anticoagulation therapy will be mentioned. This prolonged observation allowed us to capture a comprehensive view of the long-term outcomes experienced by our cohort. Furthermore, the deliberate decision to enroll patients exclusively within a single center ensured a cohesive and uniform approach to patient management, aligning with a consistent in-hospital protocol shaped collaboratively by on-site teams and tailored to the available resources.

We must acknowledge certain limitations, with the size of our cohort being a noteworthy consideration. While non-negligible, especially given the single-center enrollment, its impact on the generalizability of our findings warrants careful consideration.

## 5. Conclusions

In conclusion, our study highlights the dynamic nature of VTE management, particularly within the context of COVID-19, emphasizing the necessity for an individualized approach grounded in thorough risk assessments and collaborative efforts across diverse medical specialties and various hospital departments. The initiation of a comprehensive and well-structured diagnostic–therapeutic pathway should be preceded by an in-depth analysis of clinical characteristics, predisposing factors, and predictive indicators for mortality or adverse events associated with venous thromboembolism. Furthermore, the importance of a multidimensional evaluation, encompassing clinical, biochemical, instrumental, and functional aspects, cannot be overstated. These findings significantly contribute to an expanding body of knowledge, facilitating evidence-based decision-making in the continuously evolving landscape of VTE, both within the context of COVID-19 and beyond.

## Figures and Tables

**Table 1:** Baseline characteristics of the study population.

		Whole Cohort (*n* = 157)	VTE Associated with COVID-19 (*n* = 30)	VTE Unrelated to COVID-19 (*n* = 127)	*p*
**Age—years**		69.92 (±14.16)	66.46 (±13.57)	70.73 (±14.2)	0.138
**Male sex—*n* (%)**		78 (49.7%)	17 (56.7%)	61 (48%)	0.423
**Current smoker—*n* (%)**		21 (13.4%)	4 (13.3%)	17 (13.4%)	>0.999
**Comorbidities—*n* (%)**					
	Hypertension	85 (54.1%)	17 (56.7%)	68 (53.5%)	0.839
	Diabetes	22 (14%)	2 (6.67%)	20 (12.7%)	0.253
	Myocardial infarction	10 (6.4%)	1 (3.3%)	9 (7.1%)	0.688
	Previous VTE episode	23 (14.6%)	1 (3.3%)	22 (17.3%)	0.080
	Pulmonary hypertension	1 (0.6%)	0	1 (0.8%)	>0.999
	Chronic Cor pulmonale	0	0	0	>0.999
	Transient ischemic attack	5 (3.2%)	0	5 (3.9%)	0.584
	Atrial fibrillation	6 (3.8%)	0	6 (4.7%)	0.596
	COPD	16 (10.2%)	3 (10%)	13 (10.2%)	>0.999
	Asthma	5 (3.2%)	0	5 (3.9%)	0.584
	Interstitial lung disease	2 (1.3%)	0	2 (1.6%)	>0.999
	Peptic ulcer	3 (1.9%)	1 (3.3%)	2 (1.6%)	0.473
	Chronic kidney disease	11 (7%)	0	11 (8.7%)	0.125
	Dialysis	0	0	0	>0.999
	Anemia	9 (5.7%)	0	9 (7.1%)	0.208
	Recent transfusion	1 (0.6%)	0	1 (0.8%)	>0.999
	Chronic transfusions	0	0	0	>0.999
	Inflammatory Bowel Disease	6 (3.8%)	1 (3.3%)	5 (3.9%)	0.839
	Cancer	35 (22.3%)	4 (13.3%)	31 (24.4%)	0.229
	Antineoplastic treatment	14 (8.9%)	1 (3.3%)	13 (10.2%)	0.309
	Red Blood Cell Disorder *	4 (2.5%)	0	4 (3.2%)	>0.999
	Neuropsychiatric disorder **	16 (10.2%)	5 (16.7%)	11 (8.7%)	0.192

COPD—chronic obstructive pulmonary disease. VTE—venous thromboembolism. Recent transfusion was defined as transfusion received in the month preceding VTE diagnosis. Chronic transfusions were defined as the need for a blood transfusion on a “chronic” (or regular) basis. * Red Blood Cell Disorder was defined as any condition affecting red blood cells’ production or function, such as hemoglobinopathies. ** Neuropsychiatric Disorder was defined as any disorder affecting both the nervous system and mental health, such as depression, anxiety disorders, or schizophrenia.

**Table 2:** Baseline parameters: vital signs, VTE presentation, predictive and prognostic scores.

		Whole Cohort (*n* = 157)	VTE Associated with COVID-19 (*n* = 30)	VTE Unrelated to COVID-19 (*n* = 127)	*p*
**SBP—mmHg**		134 (±22)	138 (±13)	133 (±24)	0.202
**DBP—mmHg**		76 (±12)	79 (±9)	76 (±13)	0.345
**Heart rate—bpm**		93 (±61)	93 (±21)	94 (±66)	0.970
**Respiratory rate—bpm**		24 (±7)	28 (±7)	22 (±6)	0.006
**SO_2_—%**		94 (±6)	92 (±6)	95 (±6)	0.031
**rGENEVA—*n* (%) ^¶^**					0.581
	Low risk (0–3)	45 (29.03%)	11 (36.7%)	34 (27.2%)	
	Intermediate risk (4–10)	91 (58.71%)	16 (53.3%)	75 (60%)	
	High risk (≥11)	19 (12.26%)	3 (10%)	16 (12.8%)	
**sPESI—*n* (%) ^†^**					>0.999
	Low risk	69 (45.1%)	13 (46.4%)	56 (44.8%)	
	High risk	84 (54.9%)	15 (53.6%)	69 (55.2%)	
**PE—*n* (%) ^#^**		**126 (80.3%)**	**29 (96.7%)**	**97 (76.4%)**	0.01
	Massive PE	36 (28.6%)	5 (17.2%)	31 (32%)	
	Lobar PE	39 (31%)	9 (31%)	30 (30.9%)	
	Segmental PE	46 (36.6%)	13 (44.8%)	33 (34%)	
	Subsegmental PE	4 (3.2%)	2 (6.9%)	2 (2.1%)	
**DVT—*n* (%)**		58 (36.9%)	1 (3.4%)	57 (44.9%)	<0.001
	Lower limbs	47 (81%)	0	47 (82.5%)	
	Other DVT *	11 (19%)	1 (100%)	10 (17.5%)	
**PE with DVT—*n* (%)**		28 (17.8%)	0	28 (22%)	0.002
**Pulmonary hypertension signs at TTE—*n* (%) ^§^**		17 (18.1%)	1 (6.67%)	16 (20.2%)	0.289

SBP—systolic blood pressure; DBP—diastolic blood pressure; sPESI—simplified Pulmonary Embolism Severity Index; PE—pulmonary embolism; DVT—deep vein thrombosis; TTE—transthoracic echocardiogram. ^¶^ two missing values in the VTE unrelated to COVID-19 subgroup; ^†^ four missing values (two in each group); ^#^ one missing value in the VTE unrelated to COVID-19 subgroup; ^§^ data available in 94 patients (15 in the COVID-19 subgroup and 79 in the non-COVID-19 subgroup, respectively). The percentages are calculated based on the available data. * VTE at unusual sites, including splanchnic vein thrombosis (portal, splenic, or other) and jugular vein thrombosis.

**Table 3:** Laboratory values at baseline.

	Whole Cohort (*n*= 157)	VTE Associated with COVID-19 (*n*=30)	VTE Unrelated to COVID-19 (*n* = 127)	*p*
**Hb—g/dL**	13.7 (±9.1)	13.3 (±1.7)	13.9 (±10.2)	0.760
**Hct—%**	38.2 (±6.5)	39.3 (±5.1)	37.9 (±6.8)	0.346
**WBC—cell/mm^3^**	9469 (±3720)	9782 (±2998)	9384 (±3900)	0.605
**Platelets—cell/mm^3^**	241,712 (±90,878)	264,600 (±76,704)	235,793 (±93,582)	0.122
**INR**	1.17 (±0.16)	1.16 (±0.13)	1.17 (±0.17)	0.866
**aPTT ratio**	0.97 (±0.11)	0.99 (±0.13)	0.96 (±0.11)	0.224
**D-dimer—ng/mL**	9518 (±14,866)	8622 (±11,378)	9943 (±16,335)	0.701
**CRP—mg/L**	67.6 (±94.2)	86.7 (±102.5)	62.6 (±91.8)	0.230
**Creatinine—mg/dL**	1.02 (±0.5)	0.94 (±0.46)	1.05 (±0.52)	0.300
**ALT—U/I**	29.8 (±41.9)	29.2 (±13.9)	29.9 (±46.7)	0.931
**Bilirubin—mg/dL**	1.03 (±0.4)	0.96 (±0.1)	1.05 (±0.4)	0.099
**CK—U/I**	315 (±1986)	301 (±670)	318.64 (±2231)	0.968
**LDH—U/I**	284 (±96)	322 (±110)	274 (±90)	0.044
**hs-TnT—ng/L**	38.8 (±77.6)	17.2 (±16)	44 (±85.4)	0.232
**NTpro-BNP—ng/L**	1317 (±2283)	264 (±303)	1503 (±2436)	0.401

Hb—hemoglobin; Hct—hematocrit; WBC—white blood cells; INR—prothrombin time international normalized ratio; aPTT—activated partial thromboplastin time; CRP—C-reactive protein; ALT—alanine transaminase; CK—creatine kinase; LDH—lactic dehydrogenase; hs-TnT—high-sensitivity troponin T; NTproBNP—*n*-terminal pro b-type natriuretic peptide.

**Table 4:** Anticoagulant treatment.

	Whole Cohort (*n* = 157)	VTE Associated with COVID-19 (*n* = 30)	VTE Unrelated to COVID-19 (*n* = 127)	*p*
**First treatment**				
**LMWH—*n* (%)**	130 (79.62%)	29 (96.7%)	101 (79.53%)	0.158
**Fondaparinux—*n* (%)**	15 (9.55%)	1 (3.3%)	14 (11.02%)	
**UFH—*n* (%)**	4 (3.82%)	0	4 (3.15%)	
**DOAC—*n* (%)**	8 (5.09%)	0	8 (4.72%)	
**Treatment after first outpatient visit**				0.623 *
**LMWH—*n* (%)**	21 (13.37%)	4 (13.33%)	17 (13.38%)	
**Fondaparinux—*n* (%)**	11 (7%)	1 (3.3%)	10 (7.87%)	
**UFH—*n* (%)**	0	0	0	
**DOAC—*n* (%)**	123 (78.34%)	25 (83.33%)	98 (77.16%)	
**Warfarin—*n* (%)**	2 (1.27%)	0	2 (1.57%)	

LMWH—low-molecular-weight heparin; UFH—Unfractionated heparin; DOAC—direct oral anticoagulant. * comparison between DOAC vs. all other anticoagulants.

## Data Availability

Data collected and analyzed for the current study are available upon reasonable request and approval of the investigators and the Ethics Committee.

## Supplementary Materials

The following supporting information can be downloaded at https://www.mdpi.com/article/10.3390/jcm13061825/s1, Table S1. Predisposing factors for VTE in the study population; Table S2. Characteristics of the compression ultrasound examination assessment; Table S3. VTE BLEED score during the study time points; Table S4. Signs of pulmonary hypertension during trans-thoracic echocardiography in the first six months.
