# Supplementary material for: Venous Thromboembolism Management throughout the COVID-19 Era: Addressing Acute and Long-Term Challenges
Source: J Clin Med. 2024 Mar 21;13(6):1825. doi: 10.3390/jcm13061825 (PMC10971529; doi:10.3390/jcm13061825)
Supplement: Supplementary file 1 [file jcm-13-01825-s001.zip › jcm-2899234-supplementary.pdf]

Supplementary Materials

Table S1. Predisposing factors for VTE in the study population.

	Whole cohort (n= 157)	VTE associated with COVID-19 (n=30)	VTE unrelated to COVID-19 (n=127)	p
MAJOR risk factors				
**Fracture of the lower limbs or major trauma–** *n (%)*	3 (1.9%)	0	3 (2.4%)	>0.999
**Prior VTE (PE or DVT)–** *n (%)*	23 (14.6%)	1 (3.3%)	22 (17.3%)	0.081
**Spinal cord injury–** *n (%)*	0	0	0	>0.999
**Hospitalization due to congestive heart failure or atrial fibrillation/flutter (within 3 months)–** *n (%)*	1 (0.6%)	0	1 (0.8%)	>0.999
**Previous hip or knee replacement–** *n (%)*	0	0	0	>0.999
**Myocardial infarction (within 3 months)–** *n (%)*	0	0	0	>0.999
MODERATE risk factors				
**Arthroscopic surgery of the knee –** *n (%)*	0	0	0	>0.999
**Autoimmune disease–** *n (%)*	17 (10.8%)	1 (3.3%)	16 (12.6%)	0.076
**Blood transfusions–** *n (%)*	0	0	0	>0.999
**Central venous line–** *n (%)*	2 (1.3%)	0	2 (1.6%)	>0.999
**Intravenous catheter and electrocatheter–** *n (%)*	2 (1.3%)	1 (3.3%)	1 (0.8%)	0.346
**Chemotherapy–** *n (%)*	11 (7%)	1 (3.3%)	10 (7.9%)	0.692
**Congestive heart failure or respiratory failure –** *n (%)*	12 (7.6%)	2 (6.7%)	10 (7.9%)	>0.999
**Erythropoiesis-stimulating agents –** *n (%)*	0	0	0	>0.999
**Hormone replacement therapy–** *n (%)*	1 (0.6%)	0	1 (0.8%)	>0.999
**In vitro fertilization–** *n (%)*	1 (0.6%)	0	1 (0.8%)	>0.999
**Oral contraceptive therapy–** *n (%)*	1 (0.6%)	0	1 (0.8%)	>0.999
**Postpartum period–** *n (%)*	0	0	0	>0.999
**Infection (i.e. pneumonia, UTI)–** *n (%)*	54 (34.4%)	**27 (90%)**	**27 (21.3%)**	**<0.001**
**HIV–** *n (%)*	3 (1.9%)	0	3 (2.4%)	>0.999
**Inflammatory Bowel Disease–** *n (%)*	2 (1.3%)	1 (3.3%)	1 (0.8%)	0.346
**Cancer (highest risk in metastatic disease) –** *n (%)*	35 (22.3%)	3 (10%)	32 (25.2%)	0.089
**Paralytic stroke–** *n (%)*	0	0	0	>0.999
**Superficial vein thrombosis–** *n (%)*	4 (2.5%)	0	4 (3.1%)	>0.999
**Thrombophilia–** *n (%)*	3 (1.9%)	0	3 (2.4%)	>0.999
MINOR risk factors				
**Bed rest> 3 days–** *n (%)*	7 (4.5%)	2 (6.7%)	5 (3.9%)	0.619

**Diabetes – *n (%)***	22 (14%)	2 (6.7%)	20 (12.7%)	0.253
**Hypertension - *n (%)***	85 (54.1%)	17 (56%)	68 (53.5%)	0.839
**Immobility due to circumstances (i.e prolonged travel by plane or car)– *n (%)***	0	0	0	>0.999
**Age> 65 – *n (%)***	94 (59.9%)	17 (56.7%)	77 (60.6%)	0.685
**Laparoscopic surgery (i.e. cholecystectomy)– *n (%)***	1 (0.6%)	0	1 (0.8%)	>0.999
**Obesity– n *(%)***	20 (12.7%)	5 (16.7%)	15 (11.8%)	0.542
**Pregnancy– *n (%)***	0	0	0	>0.999
**Varicose veins– *n (%)***	12 (7.6%)	1 (3.3%)	11 (8.7%)	0.464

VTE = venous thromboembolism, PE = pulmonary embolism, DVT = deep vein thrombosis, UTI = urinary tract infection.

Table S2. Characteristics of the compression ultrasound examination assessment.

CUS	Whole cohort	VTE associated with COVID-19	VTE unrelated to COVID-19	p
**Baseline**	**n=84 (53.5%)**	**n=14 (46.67%)**	**n=70 (55.12%)**	
**No DVT**	41 (48.81%)	13 (92.86%)	28 (40%)	**0.001**
**Partial thrombosis**	13 (15.47%)	0	13 (18.57%)	
**Occlusive thrombosis**	30 (35.71%)	1 (7.14%)	29 (41.43%)	
**1 month**	**n= 27 (17.19%)**	**n=3 (10%)**	**n=24 (18.89%)**	
**No DVT**	18 (66.67%)	2 (66.66%)	16 (66.67%)	0.755
**Partial thrombosis**	6 (22.22%)	1 (33.33%)	5 (20.83%)	
**Occlusive thrombosis**	3 (11.11%)	0	3 (12.5%)	
**3 months**	**n=21 (13.37%)**	**n=3 (10%)**	**n=18 (14.17%)**	
**No DVT**	17 (80.95%)	3 (100%)	14 (77.78%)	0.662
**Partial thrombosis**	3 (14.28%)	0	3 (16.67%)	
**Occlusive thrombosis**	1 (4.76%)	0	1 (5.56%)	
**6 months**	**n=12 (7.64%)**	**n=0**	**n=12 (10.72%)**	
**No DVT**	7 (58.33%)	0	7 (58.33%)	na
**Partial thrombosis**	3 (25%)	0	3 (25%)	
**Occlusive thrombosis**	2 (16.67%)	0	2 (16.67%)	
**12 months**	**n=3 (1.91%)**	**n=0**	**n=3 (2.36%)**	
**No DVT**	1 (33.33%)	0	1 (33.33%)	na
**Partial thrombosis**	2 (66.66%)	0	2 (66.66%)	
**Occlusive thrombosis**	0	0	0	
**18 months**	**n=3 (1.91%)**	**n=0**	**n=3 (2.36%)**	
**No DVT**	2 (66.66%)	0	2 (66.66%)	na
**Partial thrombosis**	0	0	0	
**Occlusive thrombosis**	1 (33.33%)	0	1 (33.33%)	
**24 months**	**n=2 (11.76%)**	**n=0**	**n=2 (1.57%)**	
**No DVT**	1 (50%)	0	1 (50%)	na
**Partial thrombosis**	1 (50%)	0	1 (50%)	
**Occlusive thrombosis**	0	0	0	

CUS, compression ultrasound; DVT, deep vein thrombosis

Table S3. VTE BLEED score during the study time points.

	Whole cohort	VTE associated with COVID-19	VTE unrelated to COVID-19	p
Baseline	**n=157**	**n=30**	**n=127**	
**Low risk– n (%)**	82 (52.23%)	18 (60%)	64 (50.39%)	0.417
**High risk– n (%)**	75 (47.77%)	12 (40%)	63 (49.61%)	
1 month	**n=145 (92.36%)**	**n=27 (90%)**	**n=118 (92.91%)**	
**Low risk– n (%)**	76 (52.41%)	14 (51.85%)	62 (52.54%)	>0.999
**High risk– n (%)**	69 (47.59%)	13 (48.15%)	56 (47.46%)	
3 months	**n=96 (61.15%)**	**n=24 (80%)**	**n=72 (56.69%)**	
**Low risk– n (%)**	56 (58.33%)	16 (66.67%)	40 (55.56%)	0.474
**High risk– n (%)**	40 (41.67%)	8 (33.33%)	32 (44.44%)	
6 months	**n=73 (46.5%)**	**n=19 (63.33%)**	**n=54 (42.52%)**	
**Low risk– n (%)**	46 (63.01%)	16 (84.21%)	30 (55.56%)	**0.0298**
**High risk– n (%)**	27 (23.99%)	3 (15.79%)	24 (44.44%)	
12 months	**n=36 (22.92%)**	**n=7 (23.33%)**	**n=29 (22.83%)**	
**Low risk– n (%)**	23 (63.89%)	5 (71.43%)	18 (62.07%)	>0.999
**High risk– n (%)**	13 (36.11%)	2 (28.57%)	11 (37.93%)	
18 months	**n=21 (13.38%)**	**n=2 (6.67%)**	**n=19 (14.96%)**	
**Low risk– n (%)**	13 (61.9%)	2 (100%)	11 (57.9%)	0.505
**High risk– n (%)**	8 (38.1%)	0	8 (42.1%)	
24 months	**n=12 (7.64%)**	**n=2 (6.67%)**	**n=10 (7.87%)**	
**Low risk– n (%)**	7 (58.33%)	2 (100%)	5 (50%)	0.470
**High risk– n (%)**	5 (41.66%)	0	5 (50%)	

Table S4. Signs of pulmonary hypertension at trans-thoracic echocardiography in the first six-months.

	Whole cohort	VTE associated with COVID-19	VTE unrelated to COVID-19	p
**Baseline**	17/94 (18.08%)	1/15 (6.67%)	16/79 (20.25%)	0.291
**3 months**	6 /45 (13.33%)	1/15 (6.67%)	5/79 (16.67%)	0.647
**6 months**	7 /18(38.89%)	0/4	7 /14(50%)	0.119
